# Supplementary material for: Identification of photoperiod-induced specific miRNAs in the adrenal glands of Sunite sheep (Ovis aries)
Source: Front Vet Sci. 2022 Jul 22;9:888207. doi: 10.3389/fvets.2022.888207 (PMC9354845; doi:10.3389/fvets.2022.888207)
Supplement: Supplementary file 1 [file Data_Sheet_1.ZIP › Supplementary file/miRDeep2 file.zip!/miRDeep2 file/Know_Structure/SP42A3/oar-mir-10b.pdf]

miRBase precursor : oar-mir-10b
Total read count : 904283
oar-miR-10b read count : 901312
remaining reads : 2971

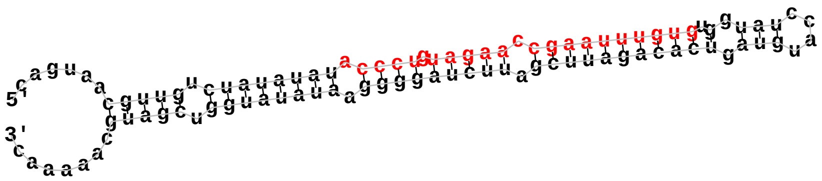

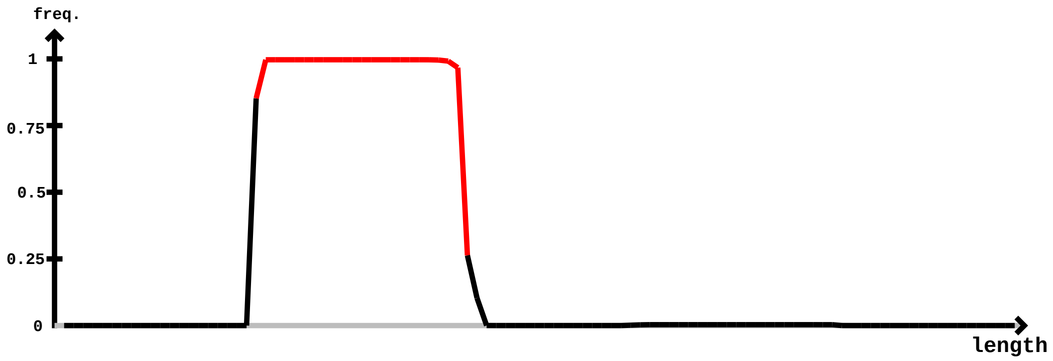


```
                    oar-miR-10b
5'- caguaacguugucuauauauacccuguagaaccgaauuugugugguauccauguagucacagauucgauucuaggggaauauauggucgaugcaaaaac   -3'   exp
    ......(((((.((((((((.((((.(((((.((((((((((.(.(((....))).))))))))))).))))))))).)))))))).))))).......    reads    mm    sample
    caguaacguugucuauau.................................................................................    1        0     seq
    ..................auacccuguagaaccgaauuug...........................................................    1        0     seq
    ..................auacccuguagaaccgaauuugu..........................................................    22       0     seq
    ..................auacccuguagaaccgaauuugug.........................................................    4        0     seq
    ...................uacccuguagaaccgaa...............................................................    12       0     seq
    ...................uacccuguagaaccgaau..............................................................    62       0     seq
    ...................uacccuguagaaccgaauu.............................................................    853      0     seq
    ...................uacccuguagaaccgaauuu............................................................    3259     0     seq
    ...................uacccuguagaaccgaauuug...........................................................    20382    0     seq
    ...................uacccuguagaaccgaauuugu..........................................................    600000   0     seq
    ...................uacccuguagaaccgaauuugug.........................................................    105741   0     seq
    ...................uacccuguagaaccgaauuugugu........................................................    40043    0     seq
    ...................uacccuguagaaccgaauuugugug.......................................................    30       0     seq
    ...................uacccuguagaaccgaauuugugugg......................................................    5        0     seq
    ...................uacccuguagaaccgaauuuguguggu.....................................................    3        0     seq
    ...................uacccuguagaaccgaauuugugugguau...................................................    1        0     seq
    ....................acccuguagaaccgaau..............................................................    8        0     seq
    ....................acccuguagaaccgaauu.............................................................    72       0     seq
    ....................acccuguagaaccgaauuu............................................................    405      0     seq
    ....................acccuguagaaccgaauuug...........................................................    1689     0     seq
    ....................acccuguagaaccgaauuugu..........................................................    36624    0     seq
    ....................acccuguagaaccgaauuugug.........................................................    38790    0     seq
    ....................acccuguagaaccgaauuugugu........................................................    53019    0     seq
    ....................acccuguagaaccgaauuugugug.......................................................    173      0     seq
    ....................acccuguagaaccgaauuugugugg......................................................    8        0     seq
    ....................acccuguagaaccgaauuuguguggu.....................................................    2        0     seq
    .....................cccuguagaaccgaauuug...........................................................    1        0     seq
    .....................cccuguagaaccgaauuugu..........................................................    6        0     seq
    .....................cccuguagaaccgaauuugug.........................................................    8        0     seq
    .....................cccuguagaaccgaauuugugu........................................................    59       0     seq
    .....................cccuguagaaccgaauuugugug.......................................................    1        0     seq
    ......................ccuguagaaccgaauuugu..........................................................    8        0     seq
    ......................ccuguagaaccgaauuugug.........................................................    9        0     seq
    ......................ccuguagaaccgaauuugugu........................................................    6        0     seq
    .......................cuguagaaccgaauuugu..........................................................    3        0     seq
```

oar-miR-10b

```
caguaacguugucuauauauacccuguagaaccgaauuugugugguauccauguagucacagauucgauucuaggggaauauauggucgaugcaaaaac
.......................cuguagaaccgaauuugug.........................................................    1     0    seq
.......................cuguagaaccgaauuugugu........................................................    3     0    seq
........................................................ucacagauucgauucuaggggaau...................    2     0    seq
.........................................................cacagauucgauucuagggga.....................    1     0    seq
.........................................................cacagauucgauucuaggggaa....................    5     0    seq
.........................................................cacagauucgauucuaggggaau...................    14    0    seq
..........................................................acagauucgauucuagggg......................    2     0    seq
..........................................................acagauucgauucuagggga.....................    22    0    seq
..........................................................acagauucgauucuaggggaa....................    47    0    seq
..........................................................acagauucgauucuaggggaau...................    1159  0    seq
..........................................................acagauucgauucuaggggaaua..................    7     0    seq
...........................................................cagauucgauucuagggga.....................    13    0    seq
...........................................................cagauucgauucuaggggaa....................    22    0    seq
...........................................................cagauucgauucuaggggaau...................    1094  0    seq
...........................................................cagauucgauucuaggggaaua..................    13    0    seq
...........................................................cagauucgauucuaggggaauau.................    3     0    seq
............................................................agauucgauucuagggga.....................    3     0    seq
............................................................agauucgauucuaggggaa....................    5     0    seq
............................................................agauucgauucuaggggaau...................    496   0    seq
............................................................agauucgauucuaggggaaua..................    30    0    seq
............................................................agauucgauucuaggggaauau.................    27    0    seq
............................................................agauucgauucuaggggaauaua................    2     0    seq
............................................................agauucgauucuaggggaauauau...............    1     0    seq
.............................................................gauucgauucuaggggaaua..................    1     0    seq
```
